# Environmental and Health Implications of the Correlation Between Arsenic and Zinc Levels in Rice from an Arsenic-Rich Zone in Cambodia

**DOI:** 10.5696/2156-9614-9.22.190603

**Published:** 2019-05-20

**Authors:** Tom Murphy, Kim Irvine, Kongkea Phan, David Lean, Ken Wilson

**Affiliations:** 1 International University, Phnom Penh, Cambodia; 2 Nanyang Technological University, Singapore; 3 Lean Environmental, Apsley, Ontario, Canada; 4 Texas State University, San Marcos, Texas, USA

**Keywords:** arsenic, zinc, rice, XRF, irrigation, drainage, fertilization, Cambodia

## Abstract

**Background.:**

In parts of Cambodia, irrigation of rice with groundwater results in arsenic accumulation in soils and rice, leading to health concerns associated with rice consumption. In Bangladesh and China, low zinc levels in rice have been found in regions where arsenic levels in rice are high. Furthermore, there have been claims that zinc deficiency is responsible for stunting of children in Cambodia. There are limited data on zinc in Cambodian rice, but in rural Asia, rice is the major source of zinc.

**Objectives.:**

To provide a preliminary evaluation of the zinc content in rice grain in Preak Russey, an area with elevated levels of arsenic. The importance of zinc in rice for infants was also assessed.

**Methods.:**

Rice cultivation was evaluated in sixty farms along the Mekong River in Cambodia. Analyses for metals, total arsenic, and arsenic species in the water and rice were conducted at the University of Ottawa, Canada by inductively coupled plasma – mass spectrometry. Analysis of total zinc and arsenic in soils were analyzed in Phnom Penh using X-ray fluorescence spectrometry (XRF). Total zinc in rice was also measured by XRF analysis.

**Results.:**

Rice in the Preak Russey area contained zinc with ½ to ¼ of the 1987 Codex standard for rice in Infant Formula. Moreover, our average zinc concentration in rice samples was less than a third that recommended for zinc fortification in rice by the United Nations World Food Programme. There was a significant (α=0.05) negative correlation between the arsenic and zinc content of rice with the lowest zinc levels occurring near the irrigation wells, the source of arsenic. There was a significantly higher content of zinc in rice from farms that fertilized with cow manure.

**Conclusions.:**

Handheld XRF spectrometers are useful tools for detection of zinc levels in rice. The potential for zinc deficiency in farmers in areas of Cambodia with arsenic toxicity is high.

**Competing Interests.:**

The authors declare no competing financial interests.

## Introduction

In 2016, the World Health Organization and the Cambodian Ministry of Health concluded that “The health of the (Cambodian) population has improved significantly… However, challenges remain including high maternal, child and neonatal mortality that continues to occur despite recent progress; malnutrition, especially in children and women; limited access to safe water and sanitation; and a growing epidemic of noncommunicable diseases and communicable diseases.”^1^ Decades ago, more than 600,000 wells were dug in Cambodia to reduce diarrhea, cholera, and other diseases initiated by drinking water from surface sources.^2^ Unfortunately, as in Bangladesh, wells in parts of Cambodia are contaminated by naturally occurring arsenic. In 1999, high concentrations of arsenic were found in Cambodian groundwater, and by 2006, the first cases of arsenicosis in Cambodia were reported.^3^ Drinking water had been the major source of arsenic. All rice passed the current Codex standards for arsenic.^4^ However the bioaccumulation of arsenic into rice via irrigation with groundwater could increase the probability of cancer.^4^,^5^ In areas of Bangladesh with clean drinking water, consumption of rice with more than 200 μg/kg of arsenic is associated with significantly higher levels of cancer, and some rice in Cambodia has more than double this threshold.^4–6^

According to one report, arsenocosis develops faster in Cambodia than expected relative to other countries with similar arsenic exposure. Arsenic inductions include congenital birth defects, suppression of mental development of children, cancer resulting in limb amputation, and death.^5^,^7^,^8^ It is thought that malnutrition might enhance arsenic toxicity in Cambodia.^3^ The leading candidate for the limiting micronutrient is zinc. Therefore, the availability of zinc should be considered in arsenic toxicity.

It has been estimated that >40% of Cambodian children are at risk of zinc deficiency.^9^ Wieringa et al. proposed that zinc deficiency in Cambodia is partially responsible for anemia and stunting in children.^10^ Greffeuille et al. estimated that 32% of Cambodian children were stunted in 2014 and proposed that zinc deficiency was a major contributing factor.^11^ This analysis is complicated by genetic hemoglobin disorders such as thalassemia which are commonly responsible for anemia and may also influence stunting.^12^ Zinc is an essential trace element required for normal growth, intellectual development, immune function, and sexual reproduction.^13^ Zinc nutrition warrants further analysis in Cambodia, especially in relation to infectious diseases, diarrheal disease, diabetes, malaria, pneumonia, linear growth retardation, and arsenic toxicity, including cancer.^14–16^ The importance of the relationship between zinc and cancer is illustrated by zinc deficiency restricting methylation and thus detoxication of arsenic.^17^ Zinc is also an essential component of superoxide dismutases. Superoxide dismutases are important in treating reactive oxygen species, which have a major role in cancer development.^18^,^19^

It has been estimated that one third of the population globally is deficient in zinc.^20^,^21^ “More than one billion people, particularly children and pregnant women suffer from zinc deficiency related health problems in Asia”.^22^ Zinc deficiency in humans reflects the fact that half of the world's soils are deficient in zinc.^23^,^24^ Zinc deficiency in soil results in decreased zinc content in crops, reduced productivity and enhanced plant disease.^25^ The cause of zinc deficiency is well understood. However, the management of zinc in rice cultivation is still evolving and does not exist in Cambodia and many other developing countries. In rural Cambodia children get most of their energy from rice which is inadequately supplemented with fish or meat. Fortified infant formula for children is only readily available in the large urban centers and there is no national fortification of rice with micronutrients. The presence of low levels of zinc in rice in areas rich in arsenic in Bangladesh and China has been previously detected, but unfortunately the concept is not yet widely recognized.^26^,^27^

**Abbreviations**
*CRM*: Certified reference material
*XRF*: X-ray fluorescence

The cultivation of rice has been optimized for centuries by empirical methods. Soil extraction and bioassay are commonly used tools requiring considerable evaluation time. There are no simple biogeochemical programs to optimize rice cultivation. Concentrations of total elements in the bulk phase of soils cannot be used to calculate bioavailable nutrients. Geochemists can measure dissolved ions, but this is expensive and technically difficult, especially in the developing world. The surface of rice roots (the rhizosphere) has a unique biogeochemical microzone. Rice plants extrude oxygen which increases the redox of this microenvironment. The transfer of oxygen from the roots to the soil varies between rice strains and likely reflects the microbial composition of the root surface.^28^ Moreover, the microbiology of the rhizosphere is not well understood. It is thought that most of the initial steps of arsenic detoxification (methylation) are mediated by microbes in the rhizosphere.^29^ Similarly, the genetics of zinc bioaccumulation vary greatly, influences how rice varieties need to be fertilized and managed, and likely also involves microbial genetics in the rhizosphere.^30^ Moreover, many rice varieties have been developed to optimize production in different soils and climates.

Genetic manipulation and plant breeding can produce rice plants with enhanced ability for zinc assimilation.^22^,^31^,^32^ Zinc enrichment is complicated, as optimal enhancement varies by rice variety, but has been successfully implemented in India to both enhance rice production and the zinc content of rice grain.^30^ Joy et al. reviewed the effective augmentation of soils in Pakistan with zinc.^23^ Soils in Pakistan have the same general derivation as in Cambodia, the runoff from the Himalayan Mountains. Attempts to enhance zinc availability by coating rice seeds with zinc were only effective in some treatments.^33^ Moreover, reviews of the effectiveness of zinc fertilization are inconsistent and fertilization is at times insufficient to alleviate zinc deficiency.^34^,^35^ The exceptions suggest that there are important regional differences in soils, rice management, and geochemistry. Pilot-scale evaluations of zinc augmentation should be implemented prior to full-scale introduction of zinc fertilizers in Cambodia.

The objective of this study is to provide a preliminary evaluation of the zinc content in rice grain in Preak Russey, an area with elevated levels of arsenic. We also compare the zinc content of rice grain to the Codex standards for zinc in Infant and Follow-Up Formula and to zinc levels in countries with national programs for micronutrient fortification of rice. In part, it is an evaluation of a phenomenon of chlorotic rice observed in an earlier study in Preak Russey and is an extension of that study.^4^The current study took advantage of the speed and simplicity of handheld X-ray fluorescence (XRF) analysis to monitor zinc concentrations in rice grain.^36^,^37^ The rice had already been collected in the earlier study.^4^,^38^

## Methods

**Figure 1:**
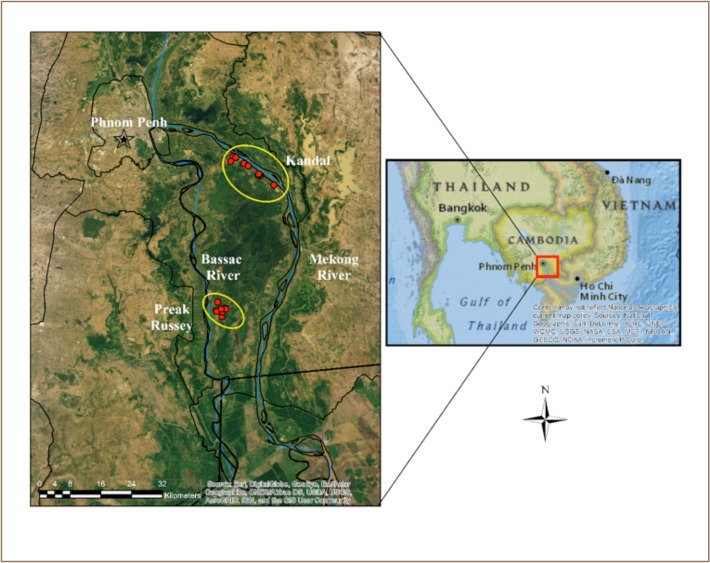
Map of the study area

**Figure 2:**
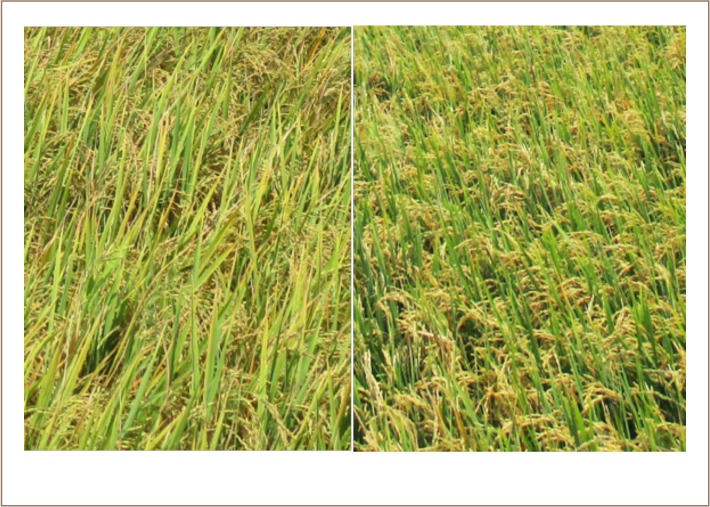
Chlorotic and healthy rice plants. Chlorotic Preak Russey-1 rice on the left and healthy Preak Russey-5 rice on the right

Figure 1 shows the study area. The arsenic content of groundwater varies from extreme highs in Preak Russey on the Bassac River to low levels of arsenic in groundwater near the Mekong River in a control site called Kandal. Both sites are in many ways very similar. The Bassac River is a distributary of the Mekong River. Both sites are mainly flood plains with farms with very similar agricultural techniques. Many of the rice samples in the present analysis were collected from farmers in 2016 as part of an earlier International Development Research Center (IDRC/CRDI)-funded project.^4^,^38^ Farmers in these areas hire combines to harvest their rice. The combines start at the outside of the field and make long loops around the edge, concentrically working towards the inside of the field. This provides partial integration of the rice. Single samples from farms Preak Russey-2 and Preak Russey-9 were collected by hand and the distances from the irrigation wells were recorded. For Preak Russey-1, Preak Russey-5 and Kandal-9, nine samples per farm were collected in a grid, equal distance apart. The Preak Russey-1 farm was the only one with chlorotic rice. The rice in Preak Russey-5 was chosen as a control to Preak Russey-1 because the rice looked healthy (greener, taller, more productive) (*Figure 2*) and both fields had a similar history of limited irrigation with groundwater (less than three years). The Preak Russey-1 and Preak Russey-5 fields were 100 m apart, grew the same rice variety (IR 85), and had similar alluvial clay soils and arsenic level in their irrigation water (~1000 μg/L arsenic).^4^,^38^

The present study mainly sampled brown rice, i.e. dehusked but not yet polished. Brown rice can be stored much longer, so it is the most common rice consumed by farmers. Rice was air dried and dehusked by hand. For the XRF analysis, rice was ground with a generic Chinese food processor (Electrical Powder Grinder DE-200 g). Between samples, tools were wiped first with a wet cloth and then with a dry cloth. Attempts to grind rice with a mortar and pestle were ineffective in producing a fine powder. The rice was ground for less than 30 seconds to avoid overheating the rice. For samples larger than 100 ml, this method worked well, but smaller samples required long delays to avoid heating the sample. We chose not to add water for grinding, but this option should be considered in the future. Adding water reduces the chance of an explosion, but would require the samples to be re-dried after grinding. Grinding freshly collected rice might be considered prior to drying. There are commercial grinders with either a centrifugal action to minimize grinding time and heating or that use liquid nitrogen to assist in grinding.^39^,^40^ The risk of a major explosion with small samples is modest, but from 1970 to 2010 there were 600 explosions in grain processing facilities in the USA with 250 fatalities and >1000 injuries.^41^

We used two different Niton XL3t GOLDD handheld XRF analyzers and a Bruker S1-600 Titan XRF analyzer for our analysis. Different XRF units were used in the present study due to availability issues. We used a two-minute analysis time with Soil Mode. All samples were processed using the sample cup method recommended by Thermo Fisher Scientific with Mylar film (*Figure 2 in Murphy et al.*).^42^ For rice, the following certified reference materials (CRMs) were used: 180–600 (soil), NIST 1568b (rice flour), CRM NIST 2710 (soil), and silica for a blank for quality assurance/quality control purposes (*Table 1*).^4^,^38^,^43^ The replication for all samples was very good, with a coefficient of variation of 6.87±7.47% for 44 samples. For four rice samples, samples were ground to a fine powder with the food processor and the results were compared to the XRF analysis of the whole grain (*Table 2*). The analysis of whole grains of rice was very consistent (average coefficient of variation 4.48±2.28%). However, analysis of the unground rice produced much higher zinc concentrations in all but one sample. The rice bran is partly a surface layer on rice grain and since the handheld XRF analyzer has weak x-rays, only the first few surface millimeters of the grain were analyzed. X-ray fluorescence analyzers have been used to measure the elemental composition of unground rice, but inductively coupled plasma optical emission spectrometry (ICP-OES) analysis was used to standardize the XRF analysis.^36^ An XRF apparatus was not available for a sufficient period of time to evaluate the variability of analysis of samples ground only with a mortar and pestle. The rice might not have needed to be ground to a fine powder. For soils, the measured mean and standard deviation of the XRF analysis of zinc in four soil CRMs (180–600, 180–646, 180–649, 180–661) were 9±12% of the certified values.^38^ The means were within 3% of the certified values for the two CRMs closest in concentration to the samples (*Table 1 in Murphy et al.*).^38^

**Table 1:** Certified Reference Material Analysis for Zinc

**Sample**	**Mean certified**	**Mean measured**
NIST 1568b (rice)	19.42±0.26	19.7±0.8
180–600 (soil)	46±3	39.7±1.5
NIST 2710 (soil)	4180±15	4203±33

All values are means of three analyses ± standard deviation.

All values are presented as μg/g.

**Table 2:** Effect of Sample Grinding on Zinc Analyses

**Sample**	**Whole grain mean**	**Powder mean**
Kd-3 March 19	29.3±0.6	19.3±1.5
PR-2 March 14	16.7±1.2	13.4±1.7
PR-2 1^st^ July 26	29.7±0.6	5.7±1.2
PR9- Mid field July 26	15±1.0	21.3±2.1

Abbreviations: Kd3, Kandal-3 farm; PR2, Preak Russey-2 farm; PR9, Preak Russey-9 farm.

Mean of three analyses ± standard deviation. All values are presented as μg/g.

Arsenic speciation of rice was performed at the University of Ottawa by inductively coupled plasma – mass spectrometry according to the United States Environmental Protection Agency (USEPA) Method 200.8.^44^ Arsenic species including arsenic(III), arsenic(V), monomethylarsonic acid and dimethylarsinic acid were quantified using the method developed by Agilent Technologies.^45^ Details can be found in earlier publications.^4^,^42^ An interview form was developed using preliminary visits to farms in the study areas in 2014 and 2015. The form was designed to quantify aspects of the farming methods and to facilitate communications and collaboration with the farmers. Details of these surveys conducted in an earlier study are reported in the Supplement to Appendix 2 of the IDRC/CRDI report and Murphy *et al.*^4^,^46^ Statistical analyses used Excel and VassarStats.^47^

## Results

**Figure 3:**
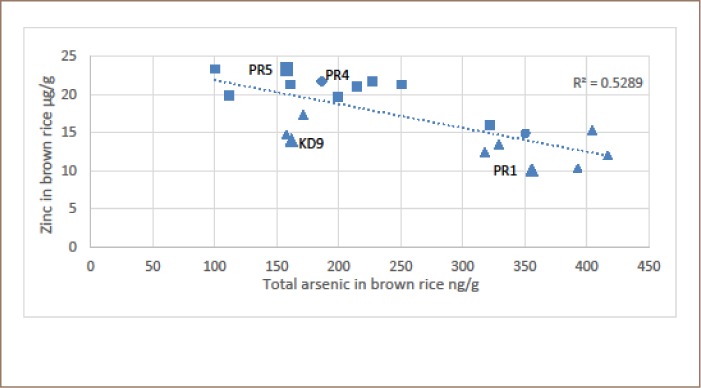
Total arsenic vs total zinc in brown rice composite samples. Squares represent farms with cows and triangles represent farms without cows. Samples represented by the larger-sized symbols (Preak Russey-1, KD-9 and Preak Russey-5) were collected with a fixed grid of nine subsamples. The diamond represents Preak Russey-4, which had a treatment ditch. Abbreviations: Kd9, Kandal-9 farm; PR5, Preak Russey-5 farm; PR4, Preak Russey-4 farm; PR1, Preak Russey-1 farm.

**Figure 4:**
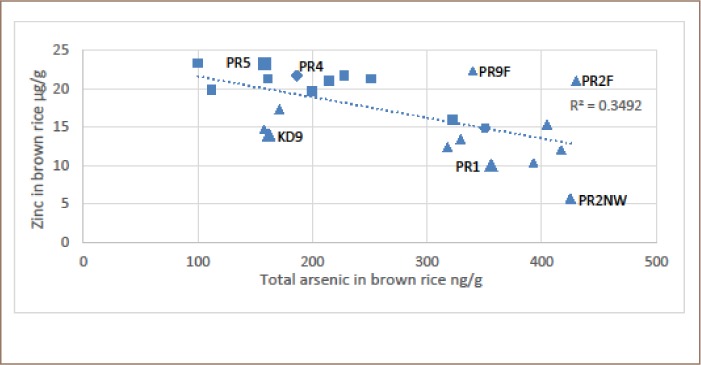
Total arsenic vs total zinc in brown rice in all samples, including individual samples that had been purposefully collected. Abbreviations: Kd9, Kandal-9 farm; PR5, Preak Russey-5 farm; PR4, Preak Russey-4 farm; PR1, Preak Russey-1 farm; PR9, Preak Russey-9 farm; PR2, Preak Russey-2 farm; NW, near well; F, far from well.

There was a significant relationship between the zinc and total arsenic content of the brown rice grain (*Figures 3 and 4*). All data were used in Figure 4. In Figure 3, three samples were excluded that were purposefully collected to reflect either close proximity to the irrigation well (Preak Russey-2 farm, near well) or furthest distance from the irrigation wells (Preak Russey-2 farm, far from well and Preak Russey-9 farm, far from well). In either case, (*Figures 3 and 4*) the R^2^ values were significant, but better when only the integrated samples (mean of the fixed grid of 9 replicates or collected by combines) were included in the analysis. The total zinc content of soils was not significantly correlated to the zinc content of the rice (data not shown). The zinc and arsenic results for site Preak Russey-1 and site Preak Russey-5 best illustrate this relationship. In both of these fields, rice and soil was sampled at 9 sites located within a fixed grid. The mean total zinc content of soil from site Preak Russey-1 and site Preak Russey-5 was 93.8±7.6 and 92.8±8.8 μg/g, respectively. By comparison, the mean total zinc of brown rice for Preak Russey-1 and Preak Russey-5 was 10.1±0.4 and 23.2±0.6 μg/g respectively (*Table 3*). Although differences in the zinc content of the rice grain were significant (t test; α=0.05), there was no significant difference in total zinc content of the soil. The rice sample with the least zinc (5.7 μg/g) came from near the irrigation well of site Preak Russey-2 where the soil had 95 μg/g of arsenic, which is about twice the arsenic level of the Dutch remediation guideline requiring consideration of intervention or remediation (55 mg/kg).^48^

**Table 3:** Analysis of Soil and Rice for Zinc

**Farm**	**Soil zinc**	**Rice zinc**
PR-1	93.8±7.6	10.1 ±0.4
PR-5	92.8±8.8	23.2±0.6

For both farms, soil and rice were collected with nine replicates in an equally spaced grid.

Abbreviations: PR1, Preak Russey-1 farm; PR5, Preak Russey-5 farm.

All values are means of three analyses ± standard deviation.

All values are presented as μg/g.

**Figure 5:**
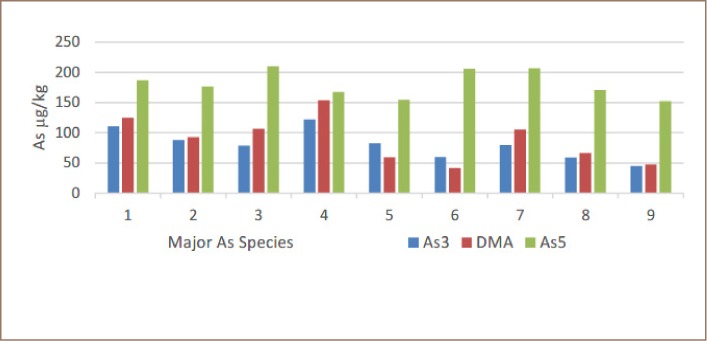
Arsenic speciation in Preak Russey-1 rice. Numbers 1–9 indicate samples collected in grid pattern equidistant from each sample. Abbreviations: As3, arsenite; DMA, dimethylarsenic acid; As5, arsenate; As, arsenic.

**Figure 6:**
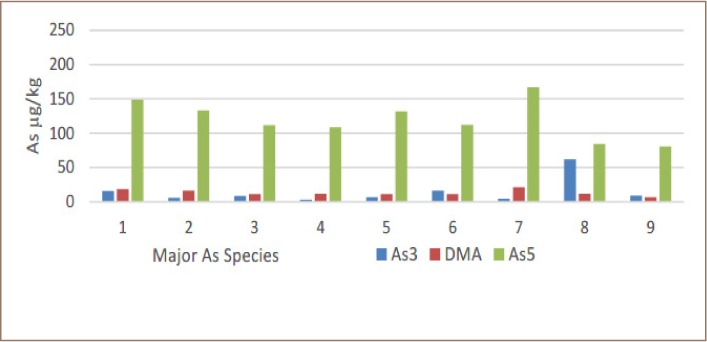
Arsenic speciation in Preak Russey-5 rice. Numbers 1–9 indicate samples collected in grid pattern equidistant from each sample. Abbreviations: As3, arsenite; DMA, dimethylarsenic acid; As5, arsenate; As, arsenic.

There are other very important differences in the rice from Preak Russey-1 and Preak Russey-5. Table 4 shows that the arsenite concentration of the rice from Preak Russey-1 was more than five times higher than equivalent rice in Preak Russey-5. The total arsenic of rice was higher in Preak Russey-1. Arsenite in rice from Preak Russey-1 was 34.6% of the inorganic arsenic, whereas in Preak Russey-5, arsenite was only 10.6% of the inorganic arsenic (*Table 2, Figures 5 and 6*).

**Table 4:** Arsenite and Total Arsenic in Preak Russey-1 and Preak Russey-5 Samples

**Site**	**Mean As^+3^**	**% As^+3^/(As^+3^+As^+5^)**	**Total arsenic**	**N**
PR-1	80.3±23.2	34.6±13	355.8±66.8	9
PR-5	14.4±18	10.6±12.4	158.5±30.3	9

Abbreviations: As^+3^, arsenite; As^+5^, arsenate; PR1, Preak Russey-1 farm; PR5, Preak Russey-5 farm; N, number of replicates.

All values are presented as μg/g.

It was observed that the rice plants of Preak Russey-1 were very different from those at the rest of the sample sites at one month prior to harvest. The leaves at Preak Russey-1 were chlorotic. The contrast to the healthy normal color of the leaves from Preak Russey-5 can be seen in Figure 2. Preak Russey-1 was the only farm with consistently chlorotic rice.

The difference in the mean zinc content of the soils between the two main sampling areas, Kandal and Preak Russey, was modest, but significant (Mann-Whitney U test, α=0.05), Kandal 78±12 μg/g, n=75 vs. Preak Russey 85±9 μg/g, n=26. As shown in both Figures 3 and 4, there were significant differences in zinc levels in rice whether or not the farmers raised cows (Mann-Whitney U test, α=0.05). The more intensive sampling of Preak Russey-5 (with cows) and Kandal-9 (no cows) supports this interpretation. Both of these farms were sampled with nine samples collected in a grid equal distance apart. Although cows are fed rice bran which is enriched in zinc (*Table 5*), the organic content of manure appears to be more important as a regulator of the zinc content of rice.

**Table 5:** Zinc Content of Rice in Preak Russey-1 and Preak Russey-5 Samples

	**Total zinc**	**N**
Brown rice	19.4±4.8	24
Polished rice	14.2±4.4	5
Rice bran	34.9±1.6	3

Abbreviation: N, number of replicates.

All values are presented as μg/g.

The mean content of zinc in groundwater irrigation water of Preak Russey farms was 3.9±4.0 μg/L. In Preak Russey, the average amount of applied irrigation water was 11,600 cubic meters per hectare. The calculated loading of zinc from irrigation is about 1% of the zinc fertilization rate suggested by the Rice Institute of 10–25 kg zinc sulfate water per ha.^49^ This concentration of zinc was close to the detection level, but the influx of zinc from irrigation with groundwater to rice was inadequate to sustain rice growth. At times the groundwater smelled like sulfides, which would precipitate zinc in the aquifer. Similarly, from the XRF analysis of eleven inorganic fertilizers and interviews with farmers, the loading of zinc in Preak Russey was estimated to be 0.15 kg/ha as hydrated zinc sulfate, which is less than 2% of the suggested fertilization rate of the Rice Institute.^49^

The zinc content of Preak Russey-4 rice was the 3rd highest observed in the present study (*Figure 3 and 4*). This farm used a treatment ditch that removed 99% of the arsenic and 92% of the iron prior to irrigation of the field. A 94% removal of arsenic and 99% removal of iron was also observed in a treatment ditch in Preak Russey-10.

## Discussion

In Asia, rice is the principal nutritional source of dietary zinc.^22^,^50^ This reflects the fact that rice is the biggest dietary component and in many rural areas, and people cannot afford to eat food that is richer in zinc, such as meat and fish. As previously mentioned, rice fed to children typically is supplemented with some fish or meat, however this supplementation is inadequate.^10^,^11^ Rice is generally believed to be the main source of zinc and it relevant to compare the zinc content of rice to major food guidelines.^22^,^50^ The current Codex standards for rice in Infant Formula or Follow-Up Formula and a similar standard proposed in 2015 for Follow-Up Formula for children 12–36 months are pertinent.^51^,^52^,^53^ These standards are expressed as 1.5 mg zinc per 100 kcal, 0.5 mg zinc per 100 kcal and 0.6 mg zinc per 100 kcal, respectively. To convert these values to the same units as zinc in rice, values were adjusted using the average energy content of rice of 3.41 kcal/g.^54^ There are slightly different energy contents for different types of rice, especially white rice, but these calculations are intended to illustrate the health concerns for zinc in brown rice at the study sites. Revisions being considered would produce Follow-Up Formula standards for children ages 6–12 months and 12–36 months. More zinc is required in infant food, but resolution must also consider the effect of additional zinc on other micronutrient absorption.

The farm that consistently had the lowest zinc content (Preak Russey-1, mean 10 μg/g, n=9) was lower than the current Codex standard for zinc in Infant Formula (44.1 μg/g), Follow-Up Formula (14.7 μg/g Codex 1987) and some international agencies have recommended this Follow-Up Formula standard be increased to 17.6 μg/g (*Table 6*).^53^ Rice in this study contained zinc with ½ to ¼ of the 1987 Codex standard for rice in Infant Formula (*Table 6*). Three farm sites (Preak Russey-1, Preak Russey-2, Preak Russey-9) at times had rice with less zinc than the Codex standard for rice in Follow-Up Formula for young children. A higher proposed zinc standard for rice in Follow-Up Formula for young children would result in one additional farm in the arsenic contaminated zone (Preak Russey-7) and two farms in the control site (Kandal-4 and Kandal-9) failing evaluation.^53^ Farms Kandal-4 and Kandal-7 had ≤10 μg/L of arsenic in their irrigation water, so although arsenic is a major factor influencing zinc bioaccumulation, it is not the only important variable, and may only be indirectly associated with the poor zinc content of rice grain. These results suggest that zinc levels in rice from outside of the arsenic zone may at times be inadequate for the development of healthy children. This is consistent with studies which have proposed that zinc deficiency is common in Cambodia.^10^ Table 6 provides several examples of zinc levels in rice.

**Table 6:** Zinc Across Studies of Brown Rice Evaluating Zinc Deficiency

**Location**	**Lowest zinc**		**Maximum zinc**	**Comments**
Cambodia	5.7^a^	10^b^	23.3	Arsenic hotspot, present study, 16 fields
Thailand^77^	20		32	Fertilization study
China^35^	20.5		24.7	Fertilization study
India^78^	26.2		67.3	65 varieties, 126 sites
India^79^	31.8		37	Fertilization study
India^80^	21.6		25.1	Fertilization study
India^81^	12.7		26.8	XRF evaluation of rice
Bangladesh^82^	29		30	Fertilization study
Bangladesh^26^	<5	13^#^	~70	Arsenic hotspot, 23 fields
Uganda^83^	14		14	Nutritional studies
Codex 1987^51^	44.1^&^			0–6 months
Codex 1987^52^	14.7^&^		NS	6–12 months
Proposed 2015^53^	17.6^&^		n.a.	12–36 months
|Proposed 2017^84^	60		n.a.	People consuming 150–300 g/d of rice

^a^integrated sample 10 m from irrigation well with soil containing 95 μg/g of arsenic

^b^mean of 9 samples from farm with chlorotic rice

^#^mean of the district with least zinc, about 4 of 220 samples from graph had ≤5 μg/g

^&^Lowest recommended concentration in Infant and Follow-Up Formula in rice foods for young children. These are all in review and these numbers are presented for comparison to rice.

Abbreviations: NS, not specified; n.a., not applicable. All units are μg/g

In a major study of zinc in Bangladesh, Williams *et al.* found consistently lower levels of zinc than our limited evaluation.^26^ Bangladesh has a longer history of using groundwater irrigation than Cambodia and it often produces more crops per year than Cambodia, both of which may enhance zinc deficiency. In 2002, Prasad stated that the severity of problems caused by zinc deficiency is not well managed or understood.^55^ Assessments of zinc deficiency are complicated and regulators need better methods of evaluating deficiency and treatment.

One of the first papers on zinc deficiency in rice stated that a rice disease in northern India was caused by zinc deficiency.^56^ Zinc deficiency reflects long periods of flooding of rice, alkaline conditions, use of high producing rice varieties, and fertilizers to boost production and multiple crops. If more irrigation water was available, a greater number of Cambodian farmers would grow a second crop and some farmers might grow a third crop. Better farm management of zinc is needed. Although improved fertilizers appear to be the most common recommendation to alleviate zinc deficiency, that is perhaps not the best solution for Preak Russey. Actual zinc demand reflects the specific field and can be higher with long periods of flooding, poor drainage, alkalinity, high arsenic or high iron, and these are all problems in Preak Russey. Reducing conditions caused by long periods of flooding inactivates zinc presumably by sulphide precipitation; thus, in reducing conditions little arsenic is required to inactivate what bioavailable zinc remains in solution.

The total zinc content of the Preak Russey soils (85±9 μg/g, n=75) and Kandal soils (78±12 μg/g, n=26) was greater than the suggested critical zinc deficiency threshold of ~10 μg/g and the suggested baseline for good zinc nutrition of soils of 60 μg/g.^26^ In addition, the zinc content of soils in the present study was similar to two areas in Bangladesh (74±17, and 97±24 zinc) where Williams et al. observed high concentrations of arsenic and low levels of zinc in rice grain.^26^ Both phosphate (an anion) and iron (a cation) can interfere with zinc bioavailability, and thus the interference is more complicated than just the ionic charge.^57^,^58^ The potential for iron interference in zinc assimilation cannot be disputed or confirmed by our study. The concentration of iron in irrigation wells in Preak Russey was 9600±6600 μg/L, n=20).^42^ Initially this iron was in solution, but it readily precipitated in the fields.^46^ The potential toxicity of iron in environments like Preak Russey has been reviewed elsewhere.^46^ However, there was no significant correlation between iron (or any other cation) in the irrigation wells and zinc in the rice grain. This sampling method may not be the most appropriate for resolving this issue. Furthermore, XRF analysis was not sensitive enough for iron in rice grain and another method of analysis should be used in future studies.

Long periods of field flooding increase soluble arsenic and iron levels, produces sulfide and precipitates zinc. However, the content of arsenic and zinc in rice grain does not only reflect the geochemistry of soils. Arsenic interferes with several metabolic pathways and zinc is an essential part of many of these same pathways in rice. The uptake, reactivity and transportation of arsenic and zinc are enhanced/mediated by metal transporters and chelators.^59–61^ The production of chelators is induced both to enhance the availability of zinc and to reduce the toxicity of arsenic. The chelator nicotianamine is primarily produced to make zinc more bioavailable, but researchers stress that other chelators like phytochelatin are mainly produced to detoxify arsenic by helping sequester it in vacuoles.^60^ Phytochelatin can also react with zinc and any imbalance in chelation created by attempts to reduce arsenic toxicity might reduce zinc availability. Raab *et al.* stated that the production of phytochelatin begins before obvious toxicity and might be a good signal of imminent suppression of productivity.^62^ In addition, chelation of metals can suppress production of reactive oxygen species which is an important means of arsenic toxicity.^63^,^64^ Many reactions occur in synchrony and an imbalance caused by arsenic toxicity may result in lower levels of zinc in the rice grain.

X-ray fluorescence analysis is currently the best protocol for monitoring zinc in rice grain in Cambodia. It is simple, fast, and inexpensive to operate. Handheld XRF units also are powered by rechargeable batteries. Unfortunately, the electrical supply in Cambodian laboratories is unreliable and damage to more complicated equipment is common and often cannot be repaired without external donor assistance.

As shown in Figure 4, the two major outliers Preak Russey-2 farm, far from well and Preak Russey-9 farm, far from well demonstrated a normal zinc content because their exposure to arsenic contaminated irrigation water was mediated by their greater distance from the well (65 m and 120 m). As demonstrated in an earlier publication, most of the arsenic precipitated in the paddy fields near the irrigation pumps (*Figures 3 and 4 in Murphy et al.*).^38^ Avoiding the use of irrigation with groundwater rich in arsenic and iron water would by itself significantly enhance the concentration of zinc in rice. The treatment ditch that some farmers used to remove arsenic from irrigation water would also accomplish this objective.^42^ The zinc content of Preak Russey-4 rice was the third highest observed in the present study (*Figure 3 and 4*). This farm used a treatment ditch that removed 99% of the arsenic and 92% of the iron prior to irrigation of the field. The removal of arsenic was likely volatilization of trimethylarsine gas.^42^ The iron is thought to have precipitated. The removal of 94% of the arsenic and 99% of the iron was also observed in a treatment ditch in Preak Russey-10, but no rice was able to be procured from that farm. At times, it was an advantage to monitor farms, not just potted rice, but some farmers were concerned about the possible negative impact to their sales and were reluctant to cooperate. Treatment ditches are a promising technique for irrigating rice with groundwater and further analysis is needed to substantiate and optimize that treatment process.

Zinc in soils is made unavailable to rice by long durations of flooding, but drainage of rice paddies can enhance the bioavailability of zinc.^65^ The critical aspects of geochemistry and seepage are not yet adequately characterized to guide the frequency of drainage or the design of ditches required to enhance zinc availability. The Preak Russey-1 site had a ditch on one side of the paddy field only, whereas the farm Preak Russey-5 that produced rice with much greater zinc content had ditches on all four sides.

There was considerable seepage from the paddy field of Preak Russey-2, but not Preak Russey-1. Preak Russey-2 had the rice sample with the least zinc (5.7 μg/g), and this sample was collected near the irrigation well where the soil had the highest level of arsenic (95 μg/g). Perhaps because this farm was slightly elevated, it had sufficient drainage to improve the bioavailability of zinc in most of the field; the rice further away from the pump had more moderate levels of zinc. The apparent stagnation in Preak Russey-1 might have resulted in greater levels of arsenic in rice proportional to the arsenic in soil in all farms in the present study (*Figure 2 in Murphy et al.*).^38^ The most relevant reflection of the importance of redox on rice in the Preak Russey-1 and Preak Russey-5 sites is the 3–4 fold higher levels of arsenite, either as a proportion of inorganic or total arsenic, respectively (*Table 4, Figures 5 and 6 in this current study, and Table 2 in the IDRC/CRDI Report*).^46^ Arsenite is more toxic than arsenate, but for the zinc geochemistry, the change in redox is the important issue.

Bhuiyan and Undan reported that the management of drainage in their study sites was mostly empirical.^65^ This also applies to Cambodia. For simple empirical management such as mid-season drainage in Cambodia, farmers need good irrigation and water storage facilities in order to assure an adequate water supply after they have drained their fields. In theory, wetlands within 5 km of Preak Russey could be used to store flood waters.^42^ Currently, farmers are unprepared to drain their fields and lose water that they may later need. Roberts and Slaton stated that in Arkansas, the only solution for severe zinc deficiency was first draining the field for two weeks, then fertilizing with zinc and nitrogen; the latter reflects the loss of nitrogen from draining the field.^66^ Applying zinc without the oxidation mediated by drainage is ineffective.^66^ Prevention is required or the cost of treatment is higher.

There are several reports of successful augmentation of soils with zinc.^23^,^30^ However, not all enrichments have been effective in both enhancing rice productivity and the zinc content of grain.^34^,^35^ Phosphorus fertilizers are known to interfere with zinc bioavailability.^58^ The apparent enhancement of zinc content of rice grain by cow manure illustrated in Figures 3 and 4 should be confirmed in a more controlled experiment. Furthermore, cow manure can both block arsenic bioaccumulation and enhance zinc bioavailability.^67^ Similarly, cow manure is known to enhance the bioavailability of refractory zinc in soils.^68^ This effect of organic matter is likely very dependent on the duration of flooding, drainage, and effect of organic matter on iron solubility and in turn on zinc solubility/adsorption.

The following analysis indicates that the manure enhancement of zinc in Preak Russey grain does not seem to reflect effective recycling of zinc. Most farms with cows had at least two adult cows and usually at least one younger animal. The typical feeding rate of rice bran was 2 kg/d per adult cow. The typical cow farmer would use about 5 kg/d of rice bran or 5 × 34.9 μg/g (bran zinc content) = 174.5 mg/d of zinc or 63692 mg zinc/year or converted to zinc sulphate (× 2.74) is 175 g zinc sulphate per year for one hectare, a typical farm size. This is less than 2% of the lowest recommended zinc dose.^49^ Unfortunately, farm productivity was not measured in the current study. The greater productivity of Preak Russey-5 relative to Preak Russey-1 is obvious in Figure 3 and future studies should evaluate the effect of enhanced zinc fertilization and any other aspect of rice management that would enhance zinc availability.

Direct fortification of rice with zinc is now commonly performed in several countries.^69^,^70^ De Pee (United Nations World Food Programme) argued that the standard for zinc in rice should be 60 μg/g, and 70 μg/g for individuals with zinc deficiency.^69^ Rice samples in our study had 28% of the recommended standard of 60 μg/g for augmented rice for the general public.^69^ Tacsan found that fortified rice in Costa Rica contains 19 μg/g of zinc.^71^ This variation likely reflects different targets of concern; small children and pregnant women require more micronutrients. Moreover, other micronutrients might be added; for example, all rice consumed in Costa Rica is fortified with folic acid, vitamins B1 (thiamin), B3 (niacin), B12 (cobalamin), E, selenium, and zinc. Folic acid would be especially relevant for parts of Cambodia where excessive amounts of arsenic are found, as folic acid is able to reduce the toxicity of arsenic.^72^

Evaluations of zinc deficiency in farmers and the effect of zinc supplementation in individuals would be complicated. Sampson found that in some farms, family members with the same food and water developed arsenicosis, but others in the same family did not.^3^ The observations of keratosis and hyperpigmentation or hypopigmentation of the skin of farmers were noteworthy in that previous analysis and in the current study.^3^ The apparent changes in sensitivity to arsenic warrant further analysis and might reflect genetic variation. Sickle cell anemia can enhance zinc deficiency and individuals with sickle cell anemia appear to benefit from zinc supplementation.^55^ Zinc deficiency might enhance arsenic toxicity. Genetic hemoglobin disease might explain some observations of arsenic toxicity in Cambodia. Sickle cell anemia is not common in Cambodia, but other forms of genetic anemia are much more common.^73^ Beta thalassemia major does not apparently enhance zinc deficiency.^74^ However, there does not appear to have been any evaluation of the effect of other common forms of genetic anemia on zinc and thus indirectly on arsenic. Hemoglobin E and alpha thalassemia are common in Cambodia. Ideally, hemoglobin genetic abnormalities should be further evaluated in a study of zinc and arsenic in Preak Russey.

Augmentation of the diet in farms can be improved, but this is complicated by zinc bioavailability in plants, including rice and beans which are often suppressed by phytates. One current practice at the best managed farm site warrants replication elsewhere. First, cows are fed rice bran. The phytase in cows' stomachs inactivates phytate and enhances zinc availability. Cow manure is then sprayed onto a fish pond that is designed to flood into the rice field. *Esomus* fish species (flying Mekong barb) have about 200 mg/kg of bioavailable zinc.^75^ Other fish species are rich in zinc, but *Esomus* species are especially zinc rich (>×2 beef) and warrant improved cultivation as they are currently popular and expensive. The bioavailability of zinc in other local foods rich in zinc such as guava leaves warrants analysis for treatment of zinc deficiency in poor rural populations.

Zinc deficiency is common globally, especially in Asia.^20^,^22^ In 2018, Wang *et al.* reported that in China, almost half of the male population was at risk of zinc deficiency, reflecting the fact that poorer populations in China getting most of their zinc from grains, including rice.^76^ Zinc deficiency in Cambodia is not unusual, but the Cambodian focus on zinc has primarily been in children.^9^,^10^ The proposed standards for zinc in children's food are three to nine times that for adults and reflects the greater zinc demand for growing children (*Table 6*). The present study of Preak Russey demonstrates that zinc deficiency seems to be associated with arsenic contamination. Children are also more susceptible to arsenic toxicity than adults. The potential for an additive effect of arsenic toxicity and zinc deficiency is high. Moreover, treating zinc deficiency might lessen arsenic toxicity. It is therefore crucial to expand upon this evaluation of zinc deficiency in areas contaminated with arsenic.

It will require several years to improve the zinc content of Cambodian rice. While agriculture is being improved, zinc supplements could correct zinc deficiency in the Cambodian diet.

## Conclusions

Handheld XRF spectrometers appear to be useful tools for detecting zinc deficiency in rice and the potential for zinc deficiency in farmers in areas of Cambodia with arsenic toxicity is high. The concentration of zinc in rice should be further evaluated in other areas of Cambodia, especially in the arsenic-contaminated zone. Empirical approaches such as irrigation with surface water (low in arsenic), soil drainage and fertilization (especially manure) should be upgraded to improve the zinc content of rice. The geochemical factors regulating the bioavailability of zinc in soils need to be better understood to guide farm management and improve the zinc content of rice. Sampling should be done with geochemical and biological measurements with reference to the distance to the source of irrigation water. Zinc deficiency in farmers, especially in the arsenic zone should be evaluated, and if confirmed, treated by improved rice cultivation, zinc fortification of rice or encouraging use of zinc sulphate supplements by farmers.
